# Potential mechanisms of cancer prevention and treatment by sulforaphane, a natural small molecule compound of plant-derived

**DOI:** 10.1186/s10020-024-00842-7

**Published:** 2024-06-21

**Authors:** Pengtao Liu, Bo Zhang, Yuanqiang Li, Qipeng Yuan

**Affiliations:** State Key Laboratory of Chemical Resource Engineering, College of Life Science and Technology, Beijing University of Chemical Technology, Beijing, P. R. China

**Keywords:** Natural small molecule compound, Sulforaphane, Cancer, Molecular mechanism, Combination therapy

## Abstract

Despite recent advances in tumor diagnosis and treatment technologies, the number of cancer cases and deaths worldwide continues to increase yearly, creating an urgent need to find new methods to prevent or treat cancer. Sulforaphane (SFN), as a member of the isothiocyanates (ITCs) family, which is the hydrolysis product of glucosinolates (GLs), has been shown to have significant preventive and therapeutic cancer effects in different human cancers. Early studies have shown that SFN scavenges oxygen radicals by increasing cellular defenses against oxidative damage, mainly through the induction of phase II detoxification enzymes by nuclear factor erythroid 2-related factor 2 (Nrf2). More and more studies have shown that the anticancer mechanism of SFN also includes induction of apoptotic pathway in tumor cells, inhibition of cell cycle progression, and suppression of tumor stem cells. Therefore, the application of SFN is expected to be a necessary new approach to treating cancer. In this paper, we review the multiple molecular mechanisms of SFN in cancer prevention and treatment in recent years, which can provide a new vision for cancer treatment.

## Introduction

According to epidemiologic data, there were 10.3 million cancer-related deaths worldwide in 2020 and 19.3 million new cancer diagnoses (Sung et al. 2021). By 2030, it is anticipated that there will be 21.6 million additional cases and 13 million fatalities (Fidler et al. 2018). Notably, despite significant advances in modern diagnostic techniques and therapeutic strategies, cancer mortality and morbidity remain high, and cancer is recognized as a significant challenge in all countries (Baig et al. 2019). Currently, practical methods for treating malignant tumors include surgery, radiotherapy, and chemotherapy agents. Among them, chemical drugs show beneficial therapeutic effects in cancer treatment. However, they also produce drug resistance and severe side effects, which finally lead to the failure of treatment (Yan et al. 2016; Wang et al. 2018). Therefore, searching for new drugs and strategies for cancer treatment remains an urgent problem.

**Fig. 1:**
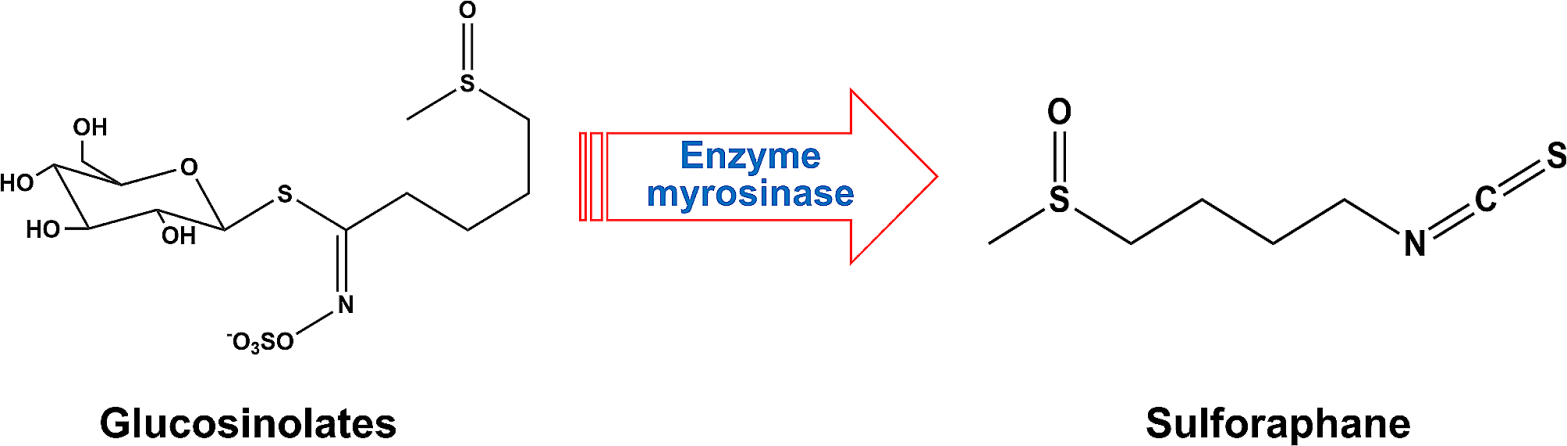
Conversion of glucosinolates to sulforaphane by hydrolysis of black mustard enzyme

A naturally occurring active substance known as isothiocyanate (ITC) originates from cruciferous vegetables, including broccoli, cabbage, and kale (Lam et al. 2009). Sulforaphane (SFN), a member of the ITCs family, is present in broccoli as glucosinolates (GLs). When broccoli is damaged, e.g., by chewing or chopping resulting in cellular fragmentation, it activates endogenous black mustard enzymes that hydrolyze the GLs and convert them to SFN (Jed et al. 2001; Bones and Rossiter 2006) (Fig. 1). SFN is a biologically active small molecule compound with anti-inflammatory and antioxidant properties (Carlos-Reyes et al. 2019; Liebman and Le 2021). SFN has been shown to play a cancer chemopreventive role by inducing phase II detoxification and antioxidant enzymes through the Nrf2/ARE signaling pathway (Brooks et al. 2001). At the same time, SFN can inhibit phase I enzymes that activate procarcinogens, thereby interfering with the initiation stage of cancer (Langoue et al. 2000). In addition to its preventive effects on cancer, SFN has recently been shown to inhibit tumor growth and progression by modulating multiple pathways associated with cancer development (Briones-Herrera et al. 2018; Negrette-Guzman 2019; Rafiei et al. 2020). Furthermore, several studies have reported that SFN can also be used as a natural dietary supplement taken with some norm chemotherapeutic medicines to increase therapeutic effectiveness while reducing their adverse side effects (Bose et al. 2018; Mielczarek et al. 2019; Xu et al. 2019; Justin et al. 2020).

**Fig. 2:**
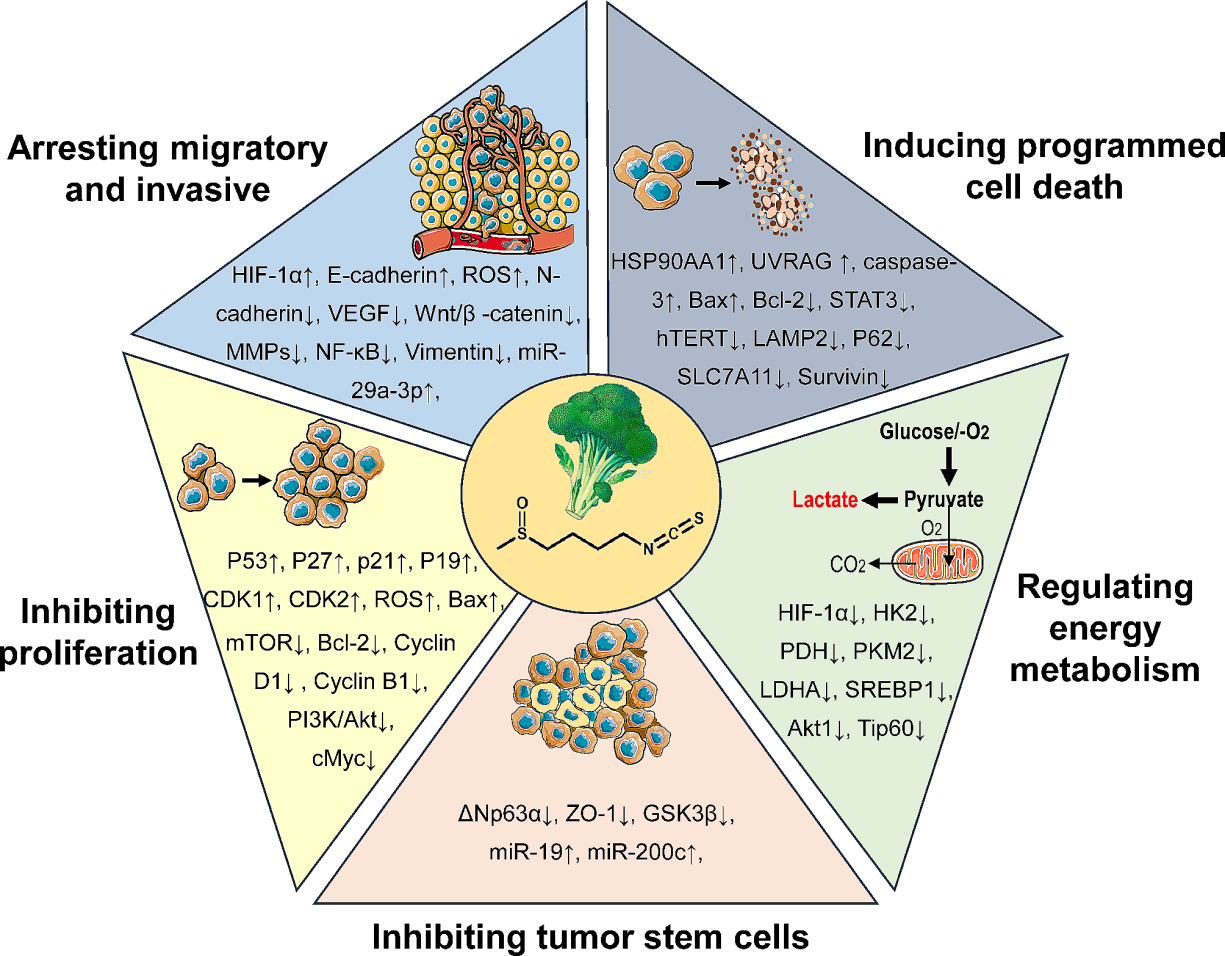
Overview of the multiple molecular mechanisms of the plant natural compound sulforaphane for cancer prevention and treatment, such as inhibiting tumor cell proliferation, arresting the invasive migration ability of tumor cells, inducing programmed cell death, inhibiting tumor stem cells, and regulating tumor cell energy metabolism

For these reasons, we reviewed the progress of research on the complex molecular mechanisms of SFN in various cancers (Fig. 2; Table 1). A better understanding of the complexity of these mechanisms will provide possible opportunities for cancer therapy. We hope this review will open new horizons for applying SFN in cancer treatment and prevention.

**Table 1:** Molecular mechanisms of cancer inhibition by sulforaphane in human cancer models. Up-arrow indicates upregulation of gene expression; downarrow indicates downregulation of gene expression

Therapy	Cancer	Dose		IC_50_	Molecular Targets	Reference	In Vitro/In Vivo
**Inhibiting proliferation**	Endometrial epithelial cancer	10 µΜ	0.75∼12.95 µΜ		Akt↓, mTOR↓, S6K↓	(Rai et al. 2020)	Both
	Osteosarcoma	20 µΜ	N/A		HDAC6↓, Akt↓, mTOR↓	(Zhang et al. 2021)	In vitro
	Ovarian cancer	2.5 ∼ 10 µM	N/A		P53↓, P27↑, Bax↑, Bcl-2↓, Cyclin-D1↓, cMyc↓, Her2↓	(Kan et al. 2018)	Both
	Gastric cancer	5 ∼ 15 µM	14.4∼20.1 µM		P53↑, P21↑	(Wang et al. 2021b)	In vitro
	Prostate cancer	1 ∼ 10 µM	5 µM		P19↑, P27↑, CDK↑, CDK2↑, CD44variants↓	(Rutz et al. 2020)	In vitro
	Cervical cancer	0 ∼ 25 µΜ	N/A		Cyclin-B1↓, GADD45β/CDC2↓	(Cheng et al. 2016)	In vitro
	Malignant gliomas	5 ∼ 50 µΜ	9.3∼35.1 µΜ		ROS↑	(Bijangi-Vishehsaraei et al. 2017)	Both
	Esophageal cancer	0 ∼ 200 µΜ	N/A		ROS↑	(Zheng et al. 2020)	In vitro
**Arresting migratory and invasive**	Gastric cancer	12.5 ∼ 100 µΜ; 10 ∼ 50 µΜ; 12 µΜ	6.31∼6.07 µΜ		HIF-1α↓, VEGF↓, AP-1↓, NF-κB↓, MMP-9↓, miR-29a-3p↑, COL3A1↓, COL5A1↓,	(Kim et al. 2015; Han et al. 2021; Li et al. 2022)	In vitro Both In vitro
	Melanoma	0–20 µΜ	N/A		MMP-2↓, MMP-9↓, TIMP3↑	(Fisher et al. 2016)	Both
	Breast cancer	0.1 ∼ 0.4 µΜ	N/A		NF-κB↓, MMP-9↓	(Zhou et al. 2022)	Both
	Glioblastoma	2.5 ∼ 70 µΜ	N/A		MMP-9↓	(Li et al. 2014; Zhang et al. 2016)	In vitro
	Prostate cancer	0 ∼ 40 µΜ	N/A		E-cadherin↑, CD44v6↓	(Peng et al. 2015)	In vitro
	Cutaneous squamous carcinoma	20 µΜ	N/A		CD44v6↓, YAP1↓	(Chen et al. 2023)	Both
	Non-small cell lung cancer	0.5 ∼ 100 µΜ	9.04∼17.35 µΜ		miR-616-5p↓, GSK3β↓, E-cadherin↑, β-cadherin↓, N-cadherin↓, Vimentin↓	(Wang et al. 2017)	Both
	Pancreatic cancer	1 ∼ 5 µΜ	9.52∼17.35 µΜ		miR-135b-5p↑, RASAL2↑	(Yin et al. 2019)	Both
	Skin squamous carcinoma	1 ∼ 16 µΜ	3.8∼11.4 µΜ		miR-199a-5p↑, Sirt1↓, CD44ICD↓	(Zhang et al. 2023)	In vitro
**Inducing programmed cell death**	Glioblastoma	0 ∼ 40 µΜ	26.6∼28.91µΜ		Caspase-3↑, Bax↑, ROS↑, Bcl-2↓, STAT3↓	(Miao et al. 2017)	In vitro
	Colorectal	10 µΜ	N/A		hTERT↓	(Martin et al. 2018)	In vitro
	Prostate cancer	0 ∼ 40 µΜ	N/A		Survivin↓	(Wiczk et al. 2012)	In vitro
		0 ∼ 20 µΜ	N/A		LAMP2↑, HSP90AA1↑, UVRAG↑	(Hahm et al. 2020)	In vitro
	Triple-negative breast cance	0 ∼ 25 µΜ	20.47 ∼ 21.93 µΜ		P62↓, Beclin1↑, LC3-II↑	(Yang et al. 2018)	Both
	Acute myeloid leukemia	0 ∼ 50 µM	N/A		GSH↓, GPX4↓	(Greco et al. 2021)	In vitro
	Small-cell lung cancer	0 ∼ 20 µM	N/A		SLC7A11↓, GSH↓	(Iida et al. 2021)	In vitro
**Inhibiting tumor stem cells**	Colorectal cancer	10 µM	N/A		ΔNp63α↓, Nanog↓, Oct4↓, Sox2↓, ZO-1↑, β-catenin↓	(Chen et al. 2022)	Both
	Lung cancer	0 ∼ 15 µΜ	N/A		miR-19↓, miR200c↑, GSK3β↓, Wnt/β-catenin↑	(Zhu et al. 2017)	Both
	Oral squamous	0 ∼ 40 µΜ	N/A		CD44↓, ALDH1↓	(Liu et al. 2017)	Both
**Regulating energy metabolism**	Bladder cancer	0 ∼ 20 µΜ	N/A		HIF-1α↓, HK2↓, PDH↓,	(Xia et al. 2019; Huang et al. 2022)	Both
	Prostate cancer	1 µΜ and 10 µM; 0 ∼ 10 µΜ; 5 ∼ 10 µM	N/A		Androgen↓, Tip60↓, HK↑, PK↑, SREBP1↓, HK2↓, PKM2↓, LDHA↓	(Singh et al. 2018, 2019; Carrasco-Pozo et al. 2019)	Both

## Regulation of tumor cell proliferative capacity by SFN

It is generally recognized that cancer cells arise from normal cells with abnormal proliferation and survival signaling pathways. They accumulate mutations during proliferation, eventually forming malignant tumor cells that can replicate indefinitely (Rycaj and Tang 2015). One of the foremost aggressive characteristics of tumor cells is proliferation, and numerous studies have shown that SFN can prevent tumor cells from developing via various processes.

The PI3K/Akt signaling pathway coordinates many physiological processes in cells, including migration, metabolism, cell viability, and proliferation (Han et al. 2018). Meanwhile, the PI3K/Akt signaling pathway promotes tumorigenesis by regulating its downstream effectors (Wang et al. 2023). Micromolar SFN concentrations inhibit endometrial epithelial tumor cell growth in vivo and in vitro by inhibiting Akt, mTOR, and ribosomal protein S6 kinase (S6K) phosphorylation (Rai et al. 2020). In human osteosarcoma cells, Zhang et al. demonstrated that SFN suppressed cell proliferation in a concentration-related way. The mechanism is that SFN inhibits cytoplasmic histone deacetylase 6 (HDAC6) and promotes Akt acetylation, decreasing the kinase catalytic activity of Akt to inhibit mTOR (Zhang et al. 2021). In conclusion, SFN would prevent tumor cell growth by regulating Akt signaling.

SFN can regulate different cancer cells’ proliferation at cell cycle stages. In human ovarian cancer, Kan et al. revealed that the SFN-treated group had considerably higher Bax, P53, and P27 expression levels. Bcl-2, Cyclin-D1, cMyc, and Her2 expression, on the other hand, were reduced in vivo and in vitro (Kan et al. 2018). According to a different study, SFN significantly increased the expression of P53 and P21 in gastric cancer cells while inhibiting the S-phase of the cell cycle and promoting apoptosis (Wang et al. 2021b). Interestingly, SFN alters the CDK-cytolytic protein axis, P19 and P27, and the expression of CD44 variations in prostate cancer cells, prompting cell cycle arrest in the S and G_2_/M phases and suppressing growth and proliferation (Rutz et al. 2020). SFN’s impact on cervical cancer cells was investigated by Cheng et al. Cyclin-B1 expression, CDC25C dephosphorylation, and remodeling of the GADD45/CDC2 complex were all restricted by SFN, which also caused a cell cycle halt in the G_2_/M phase and reduced proliferation (Cheng et al. 2016). Thus, SFN may inhibit key antitumor factors in vivo, such as P53 and P27 (Psyrri et al. 2005; Kandoth et al. 2013).

Of note, oxidative stress is upregulated in human cancers associated with elevated reactive oxygen species (ROS) and promoting DNA damage and mutation (Obtulowicz et al. 2010). In malignant gliomas, SFN inhibits tumor cell growth by inducing mitochondrial ROS accumulation and DNA damage, suppressing malignant glioma growth in vivo and in vitro (Bijangi-Vishehsaraei et al. 2017). Two studies in mice have shown that cellular senescence, an irreversible blockage of cell proliferation, is feasible as a tumor suppression strategy (Baker et al. 2016; Demaria et al. 2017). Senescence can inhibit tumorigenesis by limiting the malignant transformation of precancerous cells and blocking the proliferation of cancer cells. SFN inhibits esophageal cancer cells proliferation, triggering senescence of esophageal cancer cells by promoting ROS accumulation to induce DNA damage, modulating the mTOR/TEF3 axis to interfere with autophagy, and promoting lysosomal abnormalities (Zheng et al. 2020). Research in prostate cancer cells found that SFN induced more pronounced DNA double-strand breaks in cancer cells than in normal cells, mainly because DNA repair is more efficient in normal cells than in cancer cells, allowing SFN to inhibit cancer cell proliferation selectively in vitro (Hac et al. 2020). In summary, SFN can inhibit tumor cell proliferation by inhibiting Akt signaling, blocking the cell cycle, and inducing cellular DNA damage, providing new ideas for tumor treatment.

## Regulation of tumor cell migratory and invasive capacity by SFN

Another of the primary characteristics of many malignant tumors is migration and invasion. Cancer metastatic cells separate from the original tumor location, infiltrate mesenchymal tissues, and enter the bloodstream to start new cancer cell colonies in distant organs. Vascular endothelial growth factor (VEGF) is a crucial factor in encouraging angiogenesis, and the release of VEGF by tumor cells regulates angiogenesis in the formation of malignancies (Ahmad and Nawaz 2022). Under hypoxic conditions, VEGF is a critical protein that functions downstream of hypoxia-inducible factor-1 (HIF-1α). HIF-1α-induced up-regulation of VEGF expression activated by a hypoxic environment was inhibited by SFN, resulting in decreased migration of gastric cancer cell lines AGS and HCT116 under hypoxic conditions (Kim et al. 2015).

Elevated matrix metalloproteinase (MMP) expression in tumor cells represents higher invasion and metastasis. According to research on melanoma in vivo and in vitro, SFN therapy reduced MMP-2 and MMP-9 levels while increasing the levels of TIMP3, an MMPs inhibitor (Fisher et al. 2016). Similarly, SFN reduced invasion in gastric cancer cells by blocking the AP-1 and NF-κB signaling pathways, activated by ROS, and by reducing the expression of MMP-9 stimulated by nicotine (Li et al. 2022). Related experiments in human breast cancer cells and nude mice also demonstrated that SFN inhibited breast cancer development by inhibiting NF-κB and suppressing MMP-9 expression (Zhou et al. 2022). Additionally, because glioblastoma is a highly vascularized malignancy, SFN quickly crosses the blood-brain barrier (BBB) and builds up in the central nervous system. These effects include lowering MMP-9 release, preventing angiogenesis and invasion of glioblastoma cells, and overcoming chemoresistance (Li et al. 2014; Zhang et al. 2016).

Epithelial cells lose connection and polarity, restructure the cytoskeleton, acquire motility, and transition into the mesenchymal cellular morphology to develop an invasive phenotype. This process is known as epithelial-mesenchymal transition (EMT) (Lamouille et al. 2014). SFN inhibits the onset of EMT and invasion in advanced human prostate cancer cells by activating ERK1/2 to upregulate E-cadherin and down-regulate CD44v6 in vitro (Peng et al. 2015). Meanwhile, SFN treatment also decreased CD44v6 and YAP1 levels and their downstream genes, reducing cutaneous squamous carcinoma cell and NSG mice models’ properties and EMT markers, inhibiting cell sphere formation, invasion, and migration (Chen et al. 2023).

The processing, localization, and regulation of specific miRNAs are closely related to cancer migration and invasion (Ohtsuka et al. 2015). SFN suppresses the EMT process and the ability to metastasize non-small cell lung cancer in vivo and in vitro by altering the expression of the miR-616-5p/GSK3β/β-catenin pathway. This leads to an increase in the face of E-cadherin and a decrease in the expression of β-cadherin, N-cadherin, and vimentin (Wang et al. 2017). SFN induces overexpression of novel tumor suppressors miR-135b-5p and RASAL2 in highly aggressive pancreatic cancer cell lines and effectively inhibits tumor cell growth in vitro and in vivo (Yin et al. 2019). Similarly, SFN promoted the maturation of miR-29a-3p and inhibited the expression of COL3A1 and COL5A1 in gastric cancer cells and nude BALB/c mice. It leads to the inactivation of the downstream Wnt/β-catenin pathway, thus inhibiting gastric cancer progression (Han et al. 2021). Not only that, Zhang et al. investigated that SFN inhibited the expression of Sirt1 and CD44ICD by inducing the expression of miR-199a-5p, which in turn blocked the invasion of skin squamous carcinoma cells (Zhang et al. 2023). Therefore, developing therapies that target miRNA to inhibit cancer development and progression is an ideal strategy.

Cancer cell migration and invasion are critical reflections of the degree of tumor malignancy, and often distally migrating tumors represent a worse prognosis and a high mortality rate. Invasion and migration of cancer cells are mediated by several signaling molecules. Here, we stress the significance of SFN’s potential benefit in reducing cancer cell invasion and metastasis.

## Regulation of tumor cell programmed cell death by SFN

One of the many cell death pathways, programmed cell death (PCD), is one of the key players in many physiological and pathological states. PCD allows damaged, malignant, or no longer needed cells to be lysed and removed to maintain homeostasis within the organism (Chen et al. 2020). When PCD regulation is disturbed, it may lead to cancer, and autoimmune diseases (Fuchs and Steller 2011).

Apoptosis is an essential regulatory death mechanism in the organism by which it maintains homeostasis of its internal environment and removes unwanted, damaged, and infected cells. Most current chemotherapeutic agents induce cancer cell death through apoptosis (Carneiro and El-Deiry 2020). SFN treatment increases the levels of cleaved Caspase-3 and Bax, decreases the levels of Bcl-2, generates ROS to induce apoptosis in glioblastoma cells, and exerts anticancer effects by inhibiting the activation of the STAT3 signaling pathway in cancer cells (Miao et al. 2017). SFN significantly promotes apoptosis in glioblastoma cell lines through a mitochondria-dependent manner, manifested by cysteine asparaginase activation and DNA breaks (Sita et al. 2021). Histone deacetylase (HDAC) is overexpressed in multiple cancer subtypes. SFN affects human telomere reverse transcriptase (hTERT) mRNA levels by regulating colorectal cancer cells histone deacetylase 1 (HDAC1), which reduces hTERT protein expression and enzyme activity and induces apoptosis (Martin et al. 2018). SFN might decrease the growth of all four bladder cancer cell lines, cause apoptosis without producing toxicity, and make tumor cells more sensitive to the chemotherapy drug cisplatin, according to research by Xie et al. (Xie et al. 2022). Survivin is a critical anti-apoptotic and mitotic regulatory protein undetectable in most normal adult tissues but overexpressed in tumors. SFN inhibited prostate cancer cells growth by reducing Survivin protein synthesis by inhibiting the mTOR-S6K1-S6 signaling pathway. At the same time, SFN-induced block in protein synthesis enables cells to maintain ATP at the control cells’ level (Wiczk et al. 2012). Overall, SFN can promote apoptosis of tumor cells in various ways, exerting an inhibitory effect on cancer.

Autophagy is the process by which cells degrade substances, such as proteins, lipids, and organelles in the cytoplasm by capturing them and forming autophagosomes, which fuse with lysosomes. Autophagy is a building block and an essential energy source for cellular self-repair and maintaining homeostasis. Autophagy in cancer provides metabolic substrates for established tumors and promotes their growth (Khayati et al. 2022). It was discovered that SFN therapy boosted the levels of the crucial autophagy regulators HSP90AA1 and UVRAG as well as the lysosome-associated membrane protein 2 (LAMP2) mRNA and protein in human prostate cancer cells (Hahm et al. 2020). Additionally, SFN reduced the proliferation of TNBC cells in vitro and in vivo and triggered autophagy in a dose- and time-varying way, resulting in autophagosome development, up-regulation of Beclin1 expression, and increased synthesis of LC3-II in cancer cells (Yang et al. 2018).

An iron-dependent programmed cell death process known as ferroptosis is connected to growth blockade in many cancer cells (Zhao et al. 2020). It was found that ferroptosis induced by 50 µM SFN in acute myeloid leukemia cells were accompanied by decreased Glutathione Peroxidase 4 (GPX4) expression and increased lipid peroxidation, which provided a new extension of SFN potential as an anticancer agent (Greco et al. 2021). Iida et al. researched explored how SFN caused ferroptosis in small-cell lung cancer cells by upregulating the levels of Fe^2+^ and lipid peroxidation and downregulating the expression of glutamate anti-transporter protein xCT (SLC7A11) and total glutathione (GSH) (Iida et al. 2021). Meanwhile, SFN also increased the opening of mitochondrial permeability transition pore in gastric cancer cells. It promotes the entry of iron into mitochondria, leading to Fe^2+^ accumulation, mitochondrial dysfunction, and ferroptosis in gastric cancer cells (Wen et al. 2023).

Overall, PCD is an essential mechanism for maintaining homeostasis in the internal environment of multicellular organisms, and its role in cancer treatment is becoming more and more prominent. SFN has shown its unique characteristics in the multi-pathway regulation of PCD in cancer cells, which makes SFN a possible new strategy to inhibit tumor drug resistance.

## Combination of SFN with other anticancer drugs

Combination chemotherapy using two or more drugs with different mechanisms of action reduces drug resistance and normal cytotoxicity and is more effective than monotherapy (Zhang et al. 2015). Combination drugs have the advantage of synergistically affecting multiple survival pathways of tumor cells and can inhibit tumor heterogeneity and drug resistance (Yamada et al. 2016). SFN has anticancer activity and may also enhance the efficacy of other anticancer drugs (Table 2), such as cisplatin, clofarabine, and withaferin A in vitro (Wang et al. 2016; Royston et al. 2017).

**Table 2:** Molecular mechanisms of cancer inhibition by sulforaphane as a natural dietary supplement in combination with other anticancer drugs in human cancer models. Up-arrow indicates upregulation of gene expression; downarrow indicates downregulation of gene expression

Therapy	Cancer	Dose	IC_50_	Molecular Targets	Reference	In vitro/In vivo
**Combination of SFN with other anticancer drugs**	Breast cancer (SFN + Biochanin A)	0 ∼ 50 µΜ	27.2 µΜ	ROS↑, ERK-1/2↓	(Li et al. 2023)	In vitro
	Breast cancer (SFN + Withaferin A)	5 µΜ	N/A	Cyclin-D1↓, CDK4↓, pRB↓, E2F↑, p21↑,	(Royston et al. 2018)	In vitro
	Colorectal (SFN + Salinomycin)	0 ∼ 100 µΜ	16.97 ∼ 55.27 µΜ	PI3K↓, p-Akt↓, Bcl-2↓, Bax↑, P53↑, PARP↑	(Liu et al. 2020)	In vitro
	Melanoma (SFN + Fernblock® XP)	5 µM and 10 µM	N/A	MMPs↓	(Serini et al. 2020)	In vitro
	Ovarian cancer (SFN + Cisplatin)	0 ∼ 20 µΜ	N/A	miR-30a-3p↑	(Gong et al. 2020)	Both
	Cholangiocarcinoma (SFN + Cisplatin)	10 µΜ	N/A	Bcl-2↓, XIAP↓	(Rackauskas et al. 2017)	In vitro
	Colorectal (SFN + 5-Fluorouracil + Oxaliplatin + Calcium folinate)	2.5 ∼ 20 µΜ	N/A	MRP2↑, Bax↑, Bcl-2↑	(Čižauskaitė et al. 2022)	In vitro
	Glioblastoma (SFN + PNA-a15b)	0 ∼ 35 µΜ	N/A	miR-15b-5p↓, caspase-3↑, BAK-1↑, P53↑	(Gasparello et al. 2022b)	In vitro

In breast cancer cell lines, a two-drug combination of SFN with Biochanin A or Withaferin A induces apoptosis and inhibits cell cycle progression at lower doses (Royston et al. 2018; Li et al. 2023). In addition, the triple-agent combination of SFN, Genistein, and Nodium butyrate also showed a high degree of inhibition of breast cancer progression in vitro. The treatment resulted in a significant decrease cancer cell survival and a significant increase in apoptosis and necrosis rates with the combination treatment compared to therapy alone and the control group (Sharma and Tollefsbol 2022). Compared to control and monotherapy, treating colorectal cell lines with SFN and Salinomycin resulted in a reduction in cell migration and invasion, a boost in the number of apoptotic cells, and inhibited proliferation of colorectal cell lines in vitro and in vivo (Liu et al. 2020). The combination of SFN and the proprietary extract Fernblock® XP (FB) inhibits the production of migratory MMPs in melanoma cells and suppresses melanoma cell growth more effectively than the drug alone (Serini et al. 2020).

The combination of SFN with platinum-based drugs enhances tumor cell killing. By increasing the expression of miR-30a-3p to cause DNA damage and intracellular cisplatin concentration in cisplatin-resistant ovarian cancer cells A2780/CP70 and IGROV1-R10, Gong et al. found that SFN increased the sensitivity of ovarian cancer cells to cisplatin in vitro and in vivo (Gong et al. 2020). Combination treatment of SFN with cisplatin decreases cholangiocarcinoma cells survival, increases the cytotoxicity of cisplatin, and promotes apoptosis in a time-dependent manner (Rackauskas et al. 2017). Multidrug combination of SFN and FOLFOX (5-fluorouracil + oxaliplatin + calcium folinate) promotes the expression of multidrug resistance protein 2 (MRP2) and Bax/Bcl-2 mRNA in colorectal CX-1 cells, which is approximately 2-fold higher than that of the single-agent group (Čižauskaitė et al. 2022).

Moreover, Gasparello et al. developed a “combination therapy” based on SFN and peptide nucleic acid (PNA) targeting miRNAs. SFN and PNA-a15b (targeting miR-15b-5p) collaborative therapy induce a higher level of cellular apoptosis in glioblastoma U251 cells than using the drugs alone (Gasparello et al. 2022b). Similar findings were validated in colorectal cancer, where combination therapy of SFN with PNAs (R8-PNA-a15b, R8-PNA-a425, and R8-PNA-a584) significantly induced more apoptosis in colorectal cancer cells than either compound alone (Gasparello et al. 2020, 2022a). It suggests that combination therapy using PNA and SFN targeting tumor-associated miRNAs is a promising anticancer strategy.

In recent years, the search for natural small-molecule compounds that can effectively fight cancer has shown increasing interest. A promising anticancer method for increasing efficacy and reducing adverse effects is combining various medications with SFN.

## Other regulatory of tumors by SFN

- Regulatory of tumor stem cells by SFN.

Cancer stem cells (CSCs) have the potential for self-renewal and differentiation. They can drive cancer to regenerate repeatedly at primary and metastatic sites, employing various strategies to resist drug therapy and cell death (Batlle and Clevers 2017; Liu et al. 2019). CSCs use many of the same signaling pathways as conventional stem cells, such as the Notch, Hedgehog, and Wnt/β-catenin pathways (Song et al. 2011; Takebe et al. 2011). Since conventional therapies have shown little success in suppressing CSCs (Ehmsen et al. 2019; Molina-Pena et al. 2020; Madsen et al. 2022), finding new drugs to remove CSCs and inhibit their metastasis and drug resistance is necessary.

Several studies in recent years have found that SFN can inhibit the self-renewal of many types of CSCs (Ge et al. 2019; Wang et al. 2021a). Studies in colorectal cancer stem cell and nude mice models have shown that SFN all display a better inhibitory effect. The mechanism may regulate the expression of many stem cell markers by inhibiting ΔNp63α and upregulating ZO-1 (Chen et al. 2022). It’s interesting to note that SFN suppresses tumor stem cells by acting on miRNAs. For instance, SFN reduced the activity of oral squamous cell carcinoma CSCs and lung cancer stem cells by upregulating miR-19 and miR-200 C expression and downregulating GSK3 activation of the Wnt/β-catenin pathway. Reducing the expression of lung CSCs markers and the ability to invade and form colonies inhibited tumor growth in vivo and in vitro (Liu et al. 2017; Zhu et al. 2017). SFN significantly halts tumor progression by interfering with tumor stem cell growth, making it an ideal natural compound for cancer prevention.

- Regulation of tumor cell energy metabolism by SFN.

A characteristic feature of malignant tumor cells is their reliance on glycolysis for the energy they need to survive and thrive. Tumor cells shift their primary energy source from oxidative phosphorylation to aerobic glycolysis, a shift referred to as the Warburg effect (Schell et al. 2017).

Xia et al. showed that SFN specifically inhibited hypoxia-induced glycolysis by decreasing HIF-1α protein levels and inhibiting nuclear translocation of HIF-1α, resulting in a blocked proliferation of bladder cancer cells in vivo and in vitro (Xia et al. 2019). Another study showed that SFN also inhibited glucokinase 2 (HK2) and pyruvate dehydrogenase (PDH), decreased glycolysis and mitochondrial oxidative phosphorylation, blocked the Akt1/HK2 axis, and reduced aerobic glycolysis with abnormal glucose transport in bladder cancer cells and BBN-induced bladder tumor mouse (Huang et al. 2022). In prostate cancer, SFN has multiple mechanisms to inhibit glycolysis. SFN can inhibit androgen and Tip60-induced glycolysis in prostate cancer cells, increase the activities of HK and Pyruvate kinase (PK), and affect the proliferation and metabolism of tumor cells in vitro (Carrasco-Pozo et al. 2019). By lowering levels of the regulator of fatty acid production SREBP1, SFN can also slow the growth of prostate cancer cells and inhibition of cancer progression in the TRAMP mice model (Singh et al. 2018). Another study conducted in both prostate cancer cells and TRAMP mice have demonstrated that SFN treatment can effectively inhibit the growth of prostate cancer by reducing plasma lactate levels in the prostate and down-regulating the levels of glycolysis-related proteins HK2, pyruvate kinase isozymes M2 (PKM2), and lactate dehydrogenase A (LDHA) (Singh et al. 2019). Thus, SFN plays a significant role in targeting and improving energy metabolism in cancer patients.

## The future perspective and challenges for clinical translation of SFN

### Improving the stability of SFN in clinical applications

In recent years, the number of publications and patents on SFN inhibits cancer progression has been climbing yearly, indicating that more and more scientists worldwide are beginning to explore this fantastic small molecule compound with the hope of using it in future clinical applications. However, the problem also arises that some contradictory mechanisms inevitably appear in many SFN anticancer mechanisms. Therefore, it is imperative to fully elucidate the molecular targets of SFN and accurately characterize its efficacy. In previous studies of animal tumor models, one of the most common methods of SFN administration is injection, which is usually administered orally in clinical trials. The gap between animal studies and clinical trial scenarios requires more in-depth analyses of the anticancer effects of SFN. These are likewise impediments to the translation of SFN from basic research to clinical application. According to clinicaltrials.gov (http://ClinicalTrials.gov), SFN has conducted several cancer-related clinical trials, such as NCT03517995 (Bladder cancer, Phase 2), NCT04046653 (Prostate cancer, Not Applicable), NCT03232138 (Lung cancer, Phase 2), NCT02404428 (Prostate cancer, Not Applicable), NCT01879878 (Advanced pancreatic cancer, Not Applicable), NCT01568996 (Melanoma, Early Phase), NCT02404428 (Prostate cancer, Not Applicable), NCT01879878 (Advanced pancreatic cancer, Not Applicable), NCT01568996 (Melanoma, Early Phase 1), and so on. These clinical studies show that SFN has a favorable safety and efficacy profile in treating various cancers.

Even though impressive results have been achieved, the relatively low stability of SFN and its sensitivity to water, heat, and alkaline conditions remain significant challenges for clinical translations in the future. It makes the conditions for the production and preservation of SFN more stringent in the pharmaceutical industry or late-stage applications. Also, it hinders the translation of SFN from basic research to clinical applications. Furthermore, the bioavailability decreases after the oral administration of SFN (Soni et al. 2018). To overcome these problems, researchers have focused on improving the stability and efficiency of SFN using micro- and nanotechnology approaches. Nanotherapeutic agents can increase the bioactive concentration and improve bioavailability compared to conventional drugs (Balakumar et al. 2013). Several micro- and nano-encapsulation technologies and wall materials (e.g., hydroxypropyl-β-cyclodextrin and β-cyclodextrin), or micro- and nanocapsule technologies have been used to improve the gastrointestinal stability and bioavailability of SFN (Wu et al. 2014; Simoes et al. 2020; Yepes-Molina and Carvajal 2021). In the results of a study on human breast cancer cells, gold-coated iron oxide nanoparticles significantly improved SFN stability and activity (Manjili et al. 2016). It is also good to develop synthetic analogs of SFN and test their efficacy against cancer. Shi et al. synthesized and evaluated SFN analogs containing heterocyclic molecules. On breast cancer cells, MCF-7, SUM-159, and leukemia stem cell-like cells, KG-1a, analogs 3d, 8d, and 9d considerably outperformed SFN alone regarding their inhibitory actions (Shi et al. 2016). Georgikou et al. evaluated the cytotoxicity of seven chemically synthesized SFN analogs in three pancreatic cancer cell lines. Significant oncogenic effects of the analogs SF102 and SF134 were identified. These therapeutic effects were consistently evaluated in other tumor cell lines, suggesting that SFN analogs have potential applications in cancer therapy (Georgikou et al. 2020).

## Determining the optimal dose of SFN in clinical applications

Determining the most appropriate SFN dosage is critical to accurately guiding clinical dosing. Although most plant-derived compounds are generally safe, it is essential to analyze the toxicity of a drug before clinical trials. SFN is currently used in most preclinical studies at concentrations ranging from 0 to 40 µM, and no significant toxicity has been found. The U.S. Food and Drug Administration has limited the use of SFN to a dose of 200 µmoles in some clinical trials, and still only mild side effects such as grade 2 constipation, nausea, headache, and gastrointestinal discomfort have been observed. Still, more extreme doses of SFN have not yet been tested (Alumkal et al. 2015; Tahata et al. 2018; Zhang et al. 2020). The reported cancer progression inhibitory properties of SFN in recent years have far outweighed these mild side effects. Meanwhile, although SFN is significantly cytotoxic to cancer cells, it is relatively safe for normal cells. It has been reported that SFN inhibited the viability of about 75% of cancer cells after treating HepG2 cells with 32 µM SFN for 96 h. No significant toxicity was observed after treating human hepatocytes with 50 µM SFN for 48 h (Gross-Steinmeyer et al. 2010; Dos Santos et al. 2020). Overall, using SFN in a relatively narrow dose range is considered safe while maintaining SFN’s inhibitory effect on cancer cells. In previous studies, SFN was usually administered in a single daily dose. However, SFN is metabolized rapidly in the human body, and its half-life is very short. So far, reports on the dose-response of SFN are still scarce, and the effective dose range of SFN needs to be further determined. More high-quality studies on the dose response of SFN cancer-suppressing administration are required to provide a more accurate basis for developing rational SFN dosage regimens in clinical trials. In addition, researchers need to fill many data gaps, such as dosing frequency, contraindications, and potential adverse side effects. It can provide more detailed and accurate information for the optimal design of clinical trials, which would otherwise require a lot of time and multiple clinical trials to correct these deficiencies.

## Conclusions

Cancer has become one of humanity’s most prominent medical problems, with expensive and time-consuming treatments, low survival rates, high side effects, and a considerable burden on low and middle-income populations. Based on these problems, there is an urgent need for a simple, safe, environmentally friendly, and cost-effective novel bioactive substance that exerts maximum therapeutic efficacy while producing minimum side effects. Increasing evidence suggests that SFN exerts anti-proliferative, anti-migratory invasive, pro-apoptotic, inhibitory cell stemness and regulates energy metabolism by targeting multiple signaling pathways, including PI3K, MAPK, Wnt/β-catenin, and NF-κB, among others. Additionally, SFN, a natural dietary supplement, can effectively combine with other anticancer medications. Some preclinical studies have also shown that SFN, in combination with other anticancer drugs, can reduce the side effects of chemotherapy while exerting synergistic anticancer effects. Although the application potential, pharmacokinetic profile, and toxicity characteristics of SFN against different cancers in vivo and in vitro need to be analyzed more comprehensively and accurately, its favorable anticancer activity provides a new direction for the prevention or treatment of various cancers. Hopefully, this review will shed light on the potential value of SFN applications in cancer prevention and treatment and guide future exploration of SFN’s unknown biological functions and possible mechanisms. It would make it easier to get SFN clinically approved to treat cancer, either solely or in cooperation with other chemotherapeutic drugs.

- **Abbreviations**:

**Table:** 

ITCs	Isothiocyanates
GLs	Glucosinolates
SFN	Sulforaphane
Nrf2	Nuclear factor erythroid 2-related factor 2
GSH	Glutathione
ARE	Antioxidant response element
PI3K	Phosphoinositide 3-kinase
Akt	Protein kinase B
AMPK	Adenosine 5′-monophosphate-activated protein kinase
HDAC	Histone deacetylase
mTOR	Mechanistic target of rapamycin
S6K	Ribosomal protein S6 kinase
Bax	Bcl-2 related X protein
Bcl-2	B-cell lymphoma-2
Her2	Human epidermal growth factor receptor 2
CDK	Cyclin-dependent kinase
CDC2	Cell division cycle gene 2
CDC25C	Cell division cycle 25 homolog C
GADD45β	Growth arrest and DNA damage-inducible protein beta
ROS	Reactive oxygen species
TFE3	Transcription factor binding to IGHM enhancer 3
VEGF	Vascular endothelial growth factor
MMP	Matrix metallo proteinase
AP-1	Activator protein-1
NF-κB	Nuclear factor kappa B
BBB	Blood-brain barrier
EMT	Epithelial-mesenchymal transition
ERK	Extracellular signal-regulated kinase
E-cadherin	Epithelial cadherin
N-cadherin	Neural cadherin
YAP1	Yes-associated protein 1
RASAL2	RAS protein activator like 2
GSK3β	Glycogen synthase kinase3β
COL3A1	Collagen type III alpha 1
COL5A1	Collagen type V alpha 1
Sirt1	Silent mating type information regulation 2 homolog- 1
PCD	Programmed cell death
STAT3	Signal transducer and activator of transcription 3
hTERT	Human telomere reverse transcriptase
LAMP2	Lysosome-associated membrane protein 2
HSP90AA1	Heat shock protein 90 alpha family class a member 1
UVRAG	Ultraviolet radiation resistance-associated gene
LC3-II	Protein light chain 3 II
GPX4	Glutathione peroxidase 4
SLC7A11	Solute carrier family 7 member 11
CSCs	Cancer stem cells
ZO-1	Zonula occludens-1
HIF-1α	Hypoxia inducible factor-1α
HK	Hexokinase
PK	Pyruvate kinase
PDH	Pyruvate dehydrogenase
LDHA	Lactate dehydrogenase A
TIP60	Tat-interacting protein 60
SREBP1	Sterol regulatory element binding protein-1
ECGC	Epigallocatechin gallate
PNA	Peptide nucleic acid

## Data Availability

Not applicable.
